# Size matters for *in vitro* gene delivery: investigating the relationships among complexation protocol, transfection medium, size and sedimentation

**DOI:** 10.1038/srep44134

**Published:** 2017-03-08

**Authors:** Daniele Pezzoli, Elisa Giupponi, Diego Mantovani, Gabriele Candiani

**Affiliations:** 1 Research Unit Milano Politecnico, National Interuniversity Consortium of Materials Science and Technology – INSTM, Via Mancinelli 7, Milan 20131, Italy; 2 Laboratory for Biomaterials and Bioengineering, CRC-I, Department of Mining, Metallurgical and Materials Engineering & CHU de Quebec Research Centre, Laval University, 10 rue de l’Espinay, Quebec City (QC) G1L 3L5, Canada; 3 Department of Chemistry, Materials and Chemical Engineering “Giulio Natta”, Politecnico di Milano, Via Mancinelli 7, Milan 20131, Italy

## Abstract

Although branched and linear polyethylenimines (bPEIs and lPEIs) are gold standard transfectants, a systematic analysis of the effects of the preparation protocol of polyplexes and the composition of the transfection medium on their physicochemical behaviour and effectiveness *in vitro* have been much neglected, undermining in some way the identification of precise structure-function relationships. This work aimed to address these issues. bPEI/DNA and lPEI/DNA, prepared using two different modes of addition of reagents, gave rise to polyplexes with exactly the same chemical composition but differing in dimensions. Upon dilution in serum-free medium, the size of any kind of polyplex promptly rose over time while remained invariably stable in complete DMEM. Of note, the bigger the dimension of polyplexes (in the nano- to micrometer range), the greater their efficiency *in vitro*. Besides, centrifugal sedimentation of polyplexes displaying different dimensions to speed up and enhance their settling onto cells boosted transfection efficiencies. Conversely, transgene expression was significantly blunted in cells held upside-down and transfected, definitively pointing out the impact of gravitational sedimentation of polyplexes on their transfection efficiency. Overall, much more attention must be paid to the actual polyplex size that relies on the complexation conditions and the transfection medium.

The development of non-viral vectors capable of efficiently delivering nucleic acids to cells and tissues (*i.e.* transfection) is an inherently interdisciplinary and a rapidly advancing area of research. Since their introduction in 1987^1^, a number of cationic polymer-based vectors have been designed and developed^2^,^3^. Among them, poly(ethylene imine) (PEI) is considered the gold standard gene carrier^4^,^5^ owing to its superior transfection efficiency. PEI comes in linear (lPEI) and branched (bPEI) configurations, ranging in molecular weight from 0.8 to 1,000 kDa^6^.

Despite extensive research in this area, some conflicting results about the performances of non-viral gene delivery vectors in general, and PEIs specifically, have been reported. Such a wide range of outcomes arises, at least in part, from the large variability in experimental conditions employed in transfection assays^7^,^8^. Two major weaknesses can be identified in ordinary experimental practice that stand in the way of translating *in vitro* gene delivery results to *in vivo* efficacy studies: (i) polyplexes are nearly always characterized in unrealistic protein-free solutions disregarding the fact that, especially *in vivo*, they are meant to encounter protein-rich fluids; (ii) vectors are studied and optimized in classical two-dimensional (2D) cultures, *i.e.* with cells lying on the surface of polystyrene culture plates.

The formation of polyplexes is first driven by the electrostatic interaction between the positive charges of the cationic polymers and the negative charges of the DNA, and finally by entropy^9^,^10^. Thus, subtle changes in the way of combining reagents may affect the physicochemical properties and the transfection efficiency of polyplexes. Unfortunately, far too little is known about the influence of the mode of adding reagents on the properties of the resulting complexes, and enough detail about their preparation is very seldom acknowledged. In this regard, a comprehensive study investigating, among other possible variables, the effects on transfection activity of the order of mixing between the plasmid DNA (pDNA) and the polycationic solutions and the mixing volume ratio has been published only recently^8^. Nevertheless, it is worthy of note that some variables other than those aforementioned may also influence to some extent the way polyplexes behave.

In this context, we herein show the comparison of different modes of addition of reagents (*i.e.* the mix of reagents by pipetting vs. the dropwise addition) for the preparation of bPEI and lPEI-based polyplexes. The physicochemical properties of the resulting complexes were evaluated in each respective complexation buffer and upon dilution in serum-free and serum-supplemented media. Transfection activity was thoroughly investigated in traditional 2D upright tests and in experimental setups designed *ad-hoc* to decipher the potential effect of particle sedimentation (*i.e.* the centrifugal settling of complexes onto cells and transfections carried out in the upside-down configuration). This study allowed to outline precise size-activity relationships and to shed light on the role played by the gravitational settling of polyplexes on *in vitro* transfection outcomes, as well as to provide practical guidelines for the preparation of more and more effective polyplexes *in vitro*.

## Results and Discussion

In order to investigate the influence of differences in the complexation protocol on the physicochemical and biological properties of the resulting polyplexes, two distinct modes of addition of reagents in the preparation of PEI/pDNA polyplexes (*i.e.* the mix of reagents by pipetting, MIXING, *vs.* the dropwise addition, DROPPING) were compared in different experimental setups. Even though lPEI and bPEI have been found effective at different molecular sizes, PEIs with a *M*_*W*_ ≈ 25 kDa are by far the most studied and employed in transfection^11^,^12^, and were thus investigated in the present study.

### Physicochemical characterization of polyplexes in different complexation buffers and culture media

Commercially sourced 25 kDa bPEI and lPEI were used to complex pDNA, by adding the pDNA solution to the PEI solution, in two amongst the most popular complexation buffers such as 10 mM HEPES and 150 mM NaCl. For the sake of simplicity and comparison, polyplexes were invariably prepared at N/P 30, that was recently identified as the most effective for 25 kDa lPEI (from Polysciences)^8^. Each reagent was pre-warmed, added and incubated at 37 °C by means of a thermomixer in order to minimize temperature variations that might have some effect on complexation^13^.

The hydrodynamic diameter (*D*_*H*_) and the ζ-potential (*ζ*_*P*_) of each different kind of polyplex were first measured after dilution in its own complexation buffer. Cumulant analysis was performed for size evaluation. Z-Average and PDI, together with *ζ*_*P*_ values, are reported in Table 1. In all but one condition, that is lPEI prepared in 150 mM NaCl, the MIXING mode gave rise to the formation of cationic nanoparticles, with *ζ*_*P*_ between +22 mV and +30 mV and *D*_*H*_ between 143 and 161 nm. Of note, the PDI was invariably low (PDI < 0.2), indicating the formation of quite monodisperse particle populations. When following the DROPPING mode of addition, a significant rise in dimensions, and to a lesser extent in *ζ*_*P*_, was observed for all the conditions tested, apart from lPEI in 150 mM NaCl. Indeed, *D*_*H*_ was >350 nm and at least twice the value obtained with the MIXING protocol, while *ζ*_*P*_ was only slightly more positive. PDI increased as well (PDI > 0.4), possibly indicating the formation of heterogeneous populations of aggregates. Instead, lPEI-polyplexes in 150 mM NaCl behaved differently. Indeed, as already reported^8^,^12^, and irrespective of the mode of addition, this condition gave rise to a roughly heterogeneous population of big aggregates (PDI ≈ 1; *D*_*H*_ > 1 μm). Nonetheless, the *ζ*_*P*_ was positive and similar to the other conditions studied, albeit lower when following the DROPPING mode.

Similar results were obtained when the opposite order of mixing was used (*i.e.* PEI was added to pDNA solution) (Supp. Inf. Table S1).

Although the physicochemical behaviour of polyplexes in the complexation buffer is known to influence the final activity of such complexes^14^, their actual efficacy ultimately depends on their real properties in the cell culture medium. Unfortunately, what happens to polyplexes once diluted in various media simulating body fluids, has been much neglected and relegated mostly to descriptive, phenomenological observations. In this regard, very few studies pointed out that aggregation, stabilization or decomplexation of polyplexes may occur because of the presence of proteins that^15^,^16^, as for nanoparticles in general^17^,^18^, get adsorbed onto their surface as they get in contact with the physiological surroundings.

**Figure 1:**
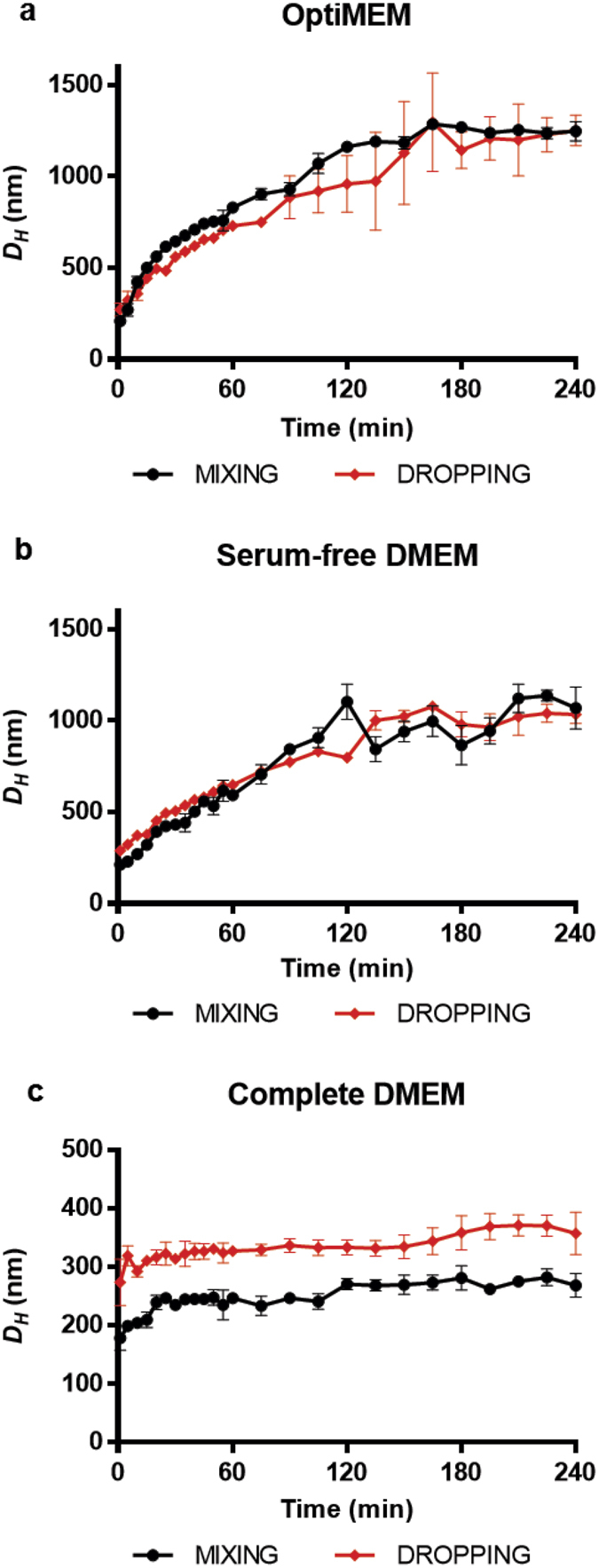
Typical temporal evolution of the *D*_*H*_ of bPEI/pDNA complexes. Polyplexes were prepared in 10 mM HEPES according to the MIXING and DROPPING modes, and diluted in (**a**) OptiMEM, (**b**) serum-free DMEM and (**c**) complete DMEM. *D*_*H*_ of polyplexes was measured by DLS, using the size distribution analysis (CONTIN algorithm) to identify the position of the main peak.

We therefore monitored the evolution of polyplex dimensions over 4 hrs at 37 °C in three different culture media. Given the broad distributions obtained for each of these conditions (PDI > 0.5), the size distribution analysis (CONTIN algorithm) was used to determine the peak positions. In the absence of serum, a general behaviour was observed, with *D*_*H*_ rising to values close to, or even higher than, 1 μm within the first 3 hrs of incubation, as exemplified in Fig. 1a and b by bPEI polyplexes prepared in 10 mM HEPES and diluted in OptiMEM^®^ I (hereinafter referred to as OptiMEM) and in plain Dulbecco’s Modified Eagle Medium (hereinafter referred to as serum-free DMEM, please see the Methods section for the formulation), respectively. Of note, one single peak corresponding to the population of polyplexes was detected in both media, thus allowing easy monitoring of temporal changes in *D*_*H*_. According to the “Derjaguin, Landau, Verwey and Overbeek theory” (DLVO theory), such a rise in dimensions may be related to an increase in electrolyte concentration^14^,^19^. Instead, the multimodal size distribution analysis of polyplexes diluted in DMEM supplemented with 10% FBS (hereinafter referred to as complete DMEM) displayed two additional and distinct peaks at 5–15 nm and 25–40 nm due to serum proteins, as confirmed by the analysis of pure complete DMEM (Supp. Inf. Fig. S1g) and in agreement with literature data^20^. Such two peaks displayed substantially steady intensities and position, and were far enough from the single sharp peak corresponding to polyplexes, so that we were able to track specifically the population of complexes all over the time course of the experiment (Supp. Inf. Fig. S1e and f, S2e and f, and S3e and f). In contrast to what we observed in serum-free media, the presence of FBS blunted the aggregation of polyplexes, as shown by the modest increase of their *D*_*H*_ of ≈50–100 nm within the first few minutes of incubation and the subsequent stabilization (Fig. 1c). We speculate that this behaviour may be due to the prompt adsorption of proteins onto polyplexes that elicited an initial increase in particle dimensions but that finally prevented long-term aggregation by reducing particle-particle interactions^14^.

Irrespective of the culture media used, no reliable results were obtained in the case of lPEI polyplexes prepared in 150 mM NaCl. It is reasonable to suppose that such aggregates became too big and polydispersed so that DLS technique is not suited to this purpose.

Altogether, these results showed that the presence of serum in the culture medium did prevent the aggregation of PEI-based polyplexes that was found to occur in serum-free media, thus allowing to keep their dimensions stable over time.

### Transfection in different culture media

**Figure 2:**
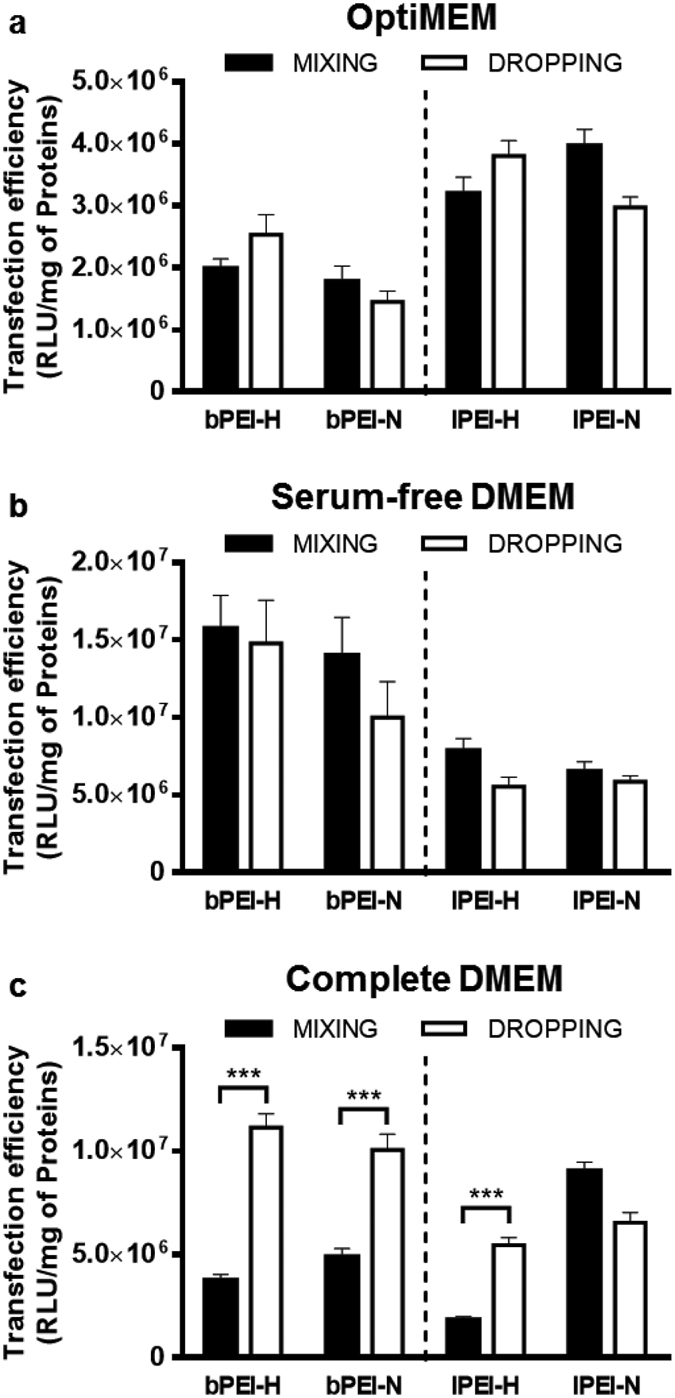
Transfection efficiency of polyplexes prepared according to different complexation protocols and tested in various transfection media on HeLa cells. Polyplexes were prepared in 10 mM HEPES (bPEI-H and lPEI-H) and 150 mM NaCl (bPEI-N and lPEI-N) at N/P 30 according to the MIXING and DROPPING modes, invariably adding pDNA to PEI solution. After dilution in (**a**) OptiMEM, (**b**) serum-free DMEM or (**c**) complete DMEM, polyplexes were added to cells and transfection efficiency was evaluated at 24 hrs. Results are expressed as mean ± SD, n ≥ 8. ***p < 0.001, MIXING *vs*. DROPPING.

To ascertain the influence of the physicochemical properties of polyplexes on gene delivery behaviour, we evaluated the transfection efficiency in every aforementioned condition (Fig. 2), keeping constant the final concentration of each component, the N/P (30), and the pDNA dose. Besides, we kept fixed the seeding density to 2 × 10^4 ^cells/cm^2^, in order to reach 60–70% confluency at the time of transfection (i.e. 24 hrs post-seeding). This condition was found to be optimal for a number of polymeric transfectants because gives rise to high effectiveness and reasonably low cytotoxicity in HeLa cell line^21^.

A common transfection behaviour was observed for all but one the different polyplexes tested in complete DMEM. Indeed, when polyplexes were prepared through the dropwise addition of reagents, their efficiency in transfections carried out in HeLa cells (Fig. 2c, *p* < 0.001 at least, MIXING *vs.* DROPPING) and in primary Human Umbilical Artery Smooth Muscle Cells (HUASMCs) (Supp. Inf. Fig. S4a, *p* < 0.01 at least, MIXING *vs.* DROPPING) doubled at least, whilst no significant variation was observed for lPEI/pDNA complexes prepared in 150 mM NaCl. Taking into account the physicochemical properties of the former complexes, a direct relationship between their dimensions and activity was unequivocally drawn: the DROPPING protocol allowed the production of bigger particles featuring higher transfection efficiency, as compared to the MIXING procedure that instead gave rise to a monodisperse population of small polyplexes (*D*_*H*_ < 200 nm). Instead, lPEI-polyplexes in 150 mM NaCl were almost not affected by the preparation protocol and invariably yielded larger polydisperse aggregates, more effective than any other PEI-buffer combination used to prepare polyplexes according to the MIXING mode. Of note, even though the overall effectiveness of lPEI/pDNA complexes prepared in 150 mM NaCl was greater as compared to the other conditions and transfectants, primary HUASMCs were generally less permissive to transfection (Supp. Inf. Fig. S4a). This cell-specific effect of a given transfectant is in line with what shown by us and other authors^7^,^22^,^23^. On the other hand, it is worth noting that the cytotoxicity in complete DMEM was invariably low in HeLa cells (between 10% and 20%, Supp. Inf. Fig. S5c), thus unaffecting the performance of the transfectants. Instead, transfection was generally slightly more detrimental to primary HUASMCs, especially in the case of highly effective lPEI/pDNA polyplexes prepared in 150 mM NaCl (Supp. Inf. Fig. S4b).

These results are particularly intriguing as they were obtained in the presence of serum that, as discussed herein above, prevented the growth in size of polyplexes and allowed to correlate the physicochemical properties measured in real conditions of use with their transfection behaviour. Indeed, it is noteworthy that serum-enriched medium is required for mid-to-long-term survival of cells in culture and is normally used to evaluate the serum-resistance of gene delivery vectors prior to animal studies^7^,^22^,^23^. However, serum-free media are also often employed in transfection, such as for the production of recombinant proteins for biopharmaceutical applications^24^,^25^. On these premises, and considering specific dimensional changes of polyplexes in serum-depleted media, we also evaluated the transfection activity of polyplexes in OptiMEM and serum-free DMEM on HeLa cells.

In contrast to what observed in complete DMEM, such stark differences in transfection efficiency observed between MIXING and DROPPING procedures decreased or even disappeared for all the conditions tested in the absence of serum (Fig. 2a and b, *p* > 0.05). These results suggested that the growth of polyplexes in OptiMEM and serum-free DMEM, as displayed in the previous section, may rapidly level out the initial differences in size leading to comparable dimensions and activity. Besides, HeLa cells transfected in both OptiMEM and serum-free DMEM displayed reduced viability as compared to complete DMEM, reaching at most 30–40% cytotoxicity (Supp. Inf. Fig. S5a and b).

Of note, a very similar behaviour on HeLa cells was also observed when the opposite order of addition (i.e. PEI was added to pDNA solution) was used to prepare polyplexes (Supp. Inf. Fig. S6).

As a general observation, transfection levels displayed in OptiMEM were lower than those in serum-free DMEM, even though both media are serum-free and have similar electrolyte content. Such unexpected outcomes may rely on the reduced cell proliferation in the former along with the protocol used for cell culture and transfection. In fact, the viability of control HeLa cells in OptiMEM was ≈23% lower than those cultivated in complete DMEM (*p* < 0.001), while no significant difference was observed between the latter condition and serum-free DMEM (Supp. Inf. Fig. S7); this issue, together with the increased cytotoxicity displayed by polyplexes (especially for bPEI) in OptiMEM, may account for impaired transfection^26^.

Altogether, these results showed the existence of a strong relationship between the size of polyplexes and their transfection efficiency *in vitro* and highlighted the key role played by the actual dimensions of PEI-based polyplexes in transfection medium on their effectiveness. It is worthy of note that the investigation of MIXING-DROPPING pairs allowed a straightforward comparison of polyplexes differing solely for size, and thus to rule out the possible effect of N/P and polymer concentration.

### Effect of centrifugation and culture in upside-down configuration on transfection efficiency

When polyplexes enter a biological *milieu*, they undergo diffusion, aggregation, and/or gravitational sedimentation^27^. As the gravitational settling rate increases with particle size and density, one possible explanation for the high efficiency of bigger complexes may rely on this. Indeed, small particles tend to stay suspended for longer periods, due to the dominant role of Brownian motion, than the larger ones^27^. Instead, bigger sized particles sediment faster, increasing the likelihood that they come into contact with cells^28^. To ascertain whether this hypothesis was true for polyplexes as well, the effectiveness of PEI/pDNA complexes was assessed in complete DMEM in two different scenarios such as their centrifugal sedimentation onto HeLa and HUASMCs, and their delivery to cells held upside-down.

**Figure 3:**
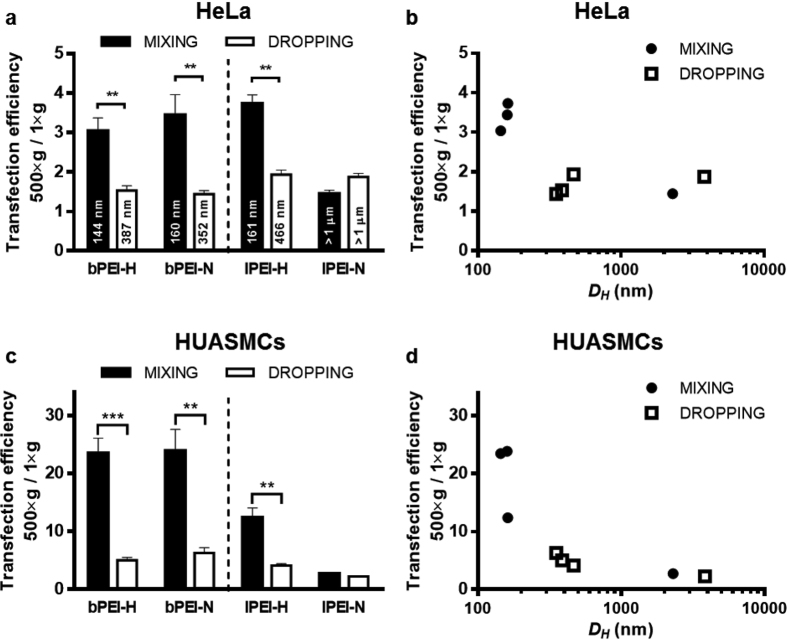
Effect of centrifugal sedimentation of polyplexes onto cells on transfection efficiency. (**a**,**c**) Ratio between transfection efficiency upon centrifugation (500 × g) of polyplexes onto (**a**) HeLa cells and (**c**) HUASMCs and under normal gravitational settling conditions (1 × g). Polyplexes were prepared in 10 mM HEPES (bPEI-H and lPEI-H) and 150 mM NaCl (bPEI-N and lPEI-N) at N/P 30 according to the MIXING and DROPPING procedures, invariably adding pDNA to PEI solution. Results are expressed as mean ± SD, n ≥ 4. **p < 0.01, ***p < 0.001, MIXING *vs.* DROPPING. (**b**,**d**) Ratio between transfection efficiency upon centrifugal sedimentation (500 × g) of polyplexes onto (**b**) HeLa cells and (**d**) HUASMCs with respect to normal gravitational conditions (1 × g) expressed in function of polyplex *D*_*H*_. n ≥ 4. Centrifugations consisted in the addition of polyplexes to (**a**,**b**) HeLa cells and (**c**,**d**) HUASMCs in complete DMEM, followed by prompt centrifugation for 5 min at 500 × g.

Centrifugation is a simple way to facilitate and speed up the settling of polyplexes onto the cell surface^4^. As expected, centrifugation increased the transfection efficiency of any kind of polyplex (Fig. 3a and c). Nonetheless, such an increase in transgene expression (Fig. 3b and d) was much greater for small polyplexes (*i.e. D*_*H*_ < 200 nm) that, on HeLa cells, did eventually become as effective as the bigger counterparts (Supp. Inf. Fig. S8a; *p* > 0.05, MIXING vs. DROPPING). Instead, whether polyplexes were big, they travelled rapidly by gravitational settling to the bottom of the well and their concentration in the vicinity of the cells was still high even without centrifugation. Again, some differences in transfection between HeLa and HUASMCs may rely on their different responsiveness to polyplexes but also on the different transfection conditions employed for the two cell types. In fact, in order to limit the cytotoxicity of polyplexes, we had to halve the dose to dispense to primary HUASMCs. We can speculate that, the lesser the polyplexes and their concentration, the lower their density at the interface with cells, and thus their transfection effectiveness under normal gravitational conditions. By the same token, the smaller the particles, the higher the increase in transfection efficiency upon centrifugal sedimentation (up to 24-fold).

Transfection experiments in the upside-down configuration were set up to shed light further on the distinct role of diffusion and gravitational settling of polyplexes on their *in vitro* activity. Of note, this setup has already been reported in other uptake studies^28^,^29^ and we showed it does not alter cellular uptake mechanisms (Supp. Inf. Fig. S9). Cells were thus seeded on glass coverslips that were turned upside-down so that the cells were facing downward, 1.5 mm-far from the bottom of the culture well (please refer to Methods).

**Figure 4:**
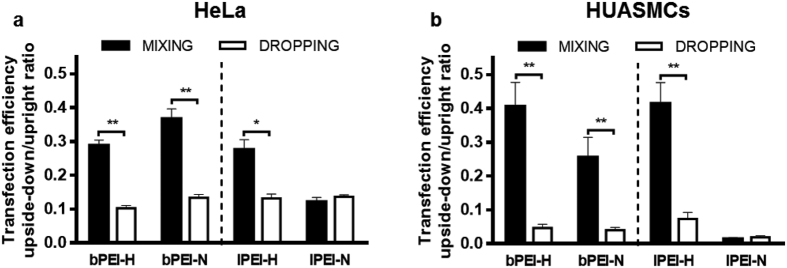
Effect of transfecting cells held upside-down on transfection efficiency. Polyplexes were prepared in 10 mM HEPES (bPEI-H and lPEI-H) and 150 mM NaCl (bPEI-N and lPEI-N) at N/P 30, according to the MIXING and DROPPING modes, invariably adding pDNA to PEI solution. Polyplexes were added to complete DMEM and delivered to (**a**) HeLa cells and to (**b**) HUASMCs held upside-down and upward. Transfection efficiency was evaluated 24 hrs post-delivery and the ratios between upside-down and upright configurations were calculated. Results are expressed as mean ± SD, n = 4. *p < 0.05, **p < 0.01, MIXING *vs.* DROPPING.

Complete DMEM-containing polyplexes was next added and the transgene activity was evaluated and compared with that of cells cultured on similar glass coverslips that were instead kept upright. One would expect that the disparity in transfection efficiency between these two configurations should be negligible if diffusion prevails over sedimentation. As shown in Fig. 4, cells invariably displayed a significant reduction of the transfection efficiency when held upside-down. Nevertheless, two different behaviours were observed: for smaller polyplexes (*i.e. D*_*H*_ < 200 nm, all the MIXING samples except for lPEI-N) the reduction of transgene expression was between 63% and 72% on HeLa cells and between 60% and 75% on HUASMCs, while for polyplexes with *D*_*H*_ > 300 nm a greater decrease (≈90% on HeLa cells and up to 98% on HUASMCs) was observed (Fig. 4; *p* < 0.05 at least, MIXING *vs.* DROPPING). As a result, the transfection activities of smaller and bigger polyplexes were mostly levelled out (Supp. Inf. Fig. 10a and c) and the cytotoxicity was low (Supp. Inf. Fig. S10b and d). These results were supported by experiments in upside-down configuration performed on HeLa cells using plasmid encoding EGFP by means of bPEI-H (Supp. Inf. Fig. S11a–d) and lPEI-N (Supp. Inf. Fig. S11e–h) polyplexes.

Whatever the PEI/pDNA complexes tested, these findings show that diffusive motion alone accounts for at most 30% of the overall activity in the case of small polyplexes (*D*_*H*_ < 200 nm), and only for 10% or less in the case of the bigger ones. Likewise, such data provide compelling evidence that gravitational settling takes part in determining the *in vitro* effectiveness of a given polymeric gene delivery vector, or otherwise said, the actual size of polyplexes influences their sedimentation and thus their transfection efficiency.

The use of small, nanometric gene delivery complexes is considered essential for effective *in vivo* delivery, because of the physical constrains such as the dimensions of the capillary fenestrations, free diffusion through tissues and the elimination from the circulation by the reticuloendothelial system (RES), that limit the efficacy of large particles^30^. However, much less attention has been paid to the effects of particle dimensions *in vitro*, where such barriers do not exist and complexes are delivered onto a cell monolayer. This fact may account for the lack of agreement between *in vitro* studies but also for some poor correlation between *in vitro* and *in vivo* outcomes.

The results reported hereinabove suggest adopting special prudence when dealing with non-viral gene delivery vectors *in vitro*, especially in the choice of the complexation protocol, the transfection medium and, more in general, the transfection setup. For instance, whereas transfections with targeted gene delivery vectors are often carried out in serum-free medium^31^,^32^,^33^, the real dimensions of polyplexes in physiological surrounding are usually neglected. In light of our results, to ascertain the effectiveness of a targeting moiety, it would be reasonable to perform experiments in the presence of serum to minimize the aggregation of single polyplexes into larger complexes, and placing cells upside-down in order to rule out the effects of gravitational settling on transfection.

## Conclusions

Herein we have demonstrated that subtle changes in the method of addition of reagents markedly influenced the physicochemical properties of PEI-polyplexes, and highlighted a strict relationship between the particle dimensions (*i.e.* their gravitational settling) and their transfection efficiency. Noteworthy, the results reported herein above suggest that, for a given gene delivery vector such as bPEI and lPEI, the bigger the dimension of polyplexes (in the nano- to micrometer range), the higher and faster their sedimentation and the greater their efficiency *in vitro*.

The dropwise addition of reagents at the complexation stage (DROPPING mode), as compared to the mix of reagents after single addition (MIXING mode), led to the formation of bigger particles, roughly stable over time, featuring higher transfection efficiency when diluted in serum-supplemented medium. Instead, in serum-free medium, polyplexes clustered over time to give micrometric aggregates. In such a condition, the differences in transfection efficiency observed in complete DMEM within every MIXING-DROPPING pair were completely blunted. Other experiments were designed *ad hoc* to ascertain the role of gravitational settling and diffusion of polyplexes on their transfection effectiveness. Centrifugation of polyplexes has shown to boost their settling onto cells and in this way transfection efficiency. Likewise, in experiments carried out with cells placed upside-down, the transfection efficiencies were dramatically diminished because of the gravitational sedimentation of polyplexes.

Altogether, this work highlights for the first time the need to take into account the real size of polyplexes, and the issue of sedimentation, for the *in vitro* development of transfectants. In this regard, special attention must be payed to the complexation conditions, the transfection medium and the experimental setup. We believe this will soon allow identifying reliable relationships that best explain some discrepancies between *in vitro* and *in vivo* outcomes. Because there is unfortunately no simple way to fine-tune the size of polyplexes, we propose a trick to increase their effectiveness *in vitro*. Indeed, the centrifugal sedimentation (5 min at 500 × g in complete DMEM) of polyplexes onto cells gives rise invariably to the highest transfection efficiency for every specific condition of complexation buffer and MIXING-DROPPING pair.

## Methods

### Materials

pDNA encoding the modified firefly luciferase (pGL3-Control Vector, 5.2 kbp) and Luciferase Assay System were obtained from Promega (Milan, Italy), pDNA encoding the Enhanced Green Fluorescent Protein (pEGFP-N1) was from Clontech Laboratories (Mountain View, CA, USA); pDNA purification kits were from Qiagen (Milan, Italy). Bicinchoninic acid (BCA) Protein Assay Kit, OptiMEM^®^ I, AlamarBlue cell viability reagent, Fibroblast Growth Factor-basic (FGFb), and Epidermal Growth Factor (EGF) were purchased from Life Technologies Italia (Monza, Italy) while human insulin was from Santa Cruz Biotechnology (Dallas, TX, USA).

HeLa (human epithelial ovarian carcinoma cells, CCL-2.2) cell line and HUASMCs (primary Human Umbilical Artery Smooth Muscle Cells, CC-2579) were purchased from the American Type Culture Collection (ATCC, Manassas, VA, USA) and from Lonza (Walkersville, MD, USA), respectively. 70 kDa fluorescein isothiocyanate (FITC)-dextran was from Sigma-Aldrich (Milan, Italy). 25 kDa lPEI was from Polysciences (Eppelheim, Germany) and 25 kDa bPEI was from Sigma-Aldrich (Milan, Italy), as all other chemicals if not differently specified.

### Preparation of PEI solutions

25 kDa lPEI and 25 kDa bPEI were diluted to a final concentration of 0.86 mg/mL in 10 mM HEPES buffer or 150 mM NaCl and the pH was adjusted to 7.0. The amine concentration ([N]) in such stock solutions was 20 mM, considering that there is one nitrogen per repeat unit of PEI, -NHCH_2_CH_2_-, corresponding to a molecular weight (*M*_*w*_) of 43 Da^34^.

### Preparation of polyplexes

pDNA was amplified, isolated, purified and diluted in 0.1× TE buffer (1 mM Tris, pH 8.0; 0.1 mM ethylenediaminetetraacetic acid - EDTA) as described before^8^. Polyplexes were prepared at 37 °C by combining pre-warmed PEI and pDNA solutions to give a final DNA concentration of 20 ng/μL and N/P 30, where N/P is defined as the number of amines (N) of the cationic polymer per DNA phosphate group (P) in the complexation solution. 10 mM HEPES and 150 mM NaCl, both at pH 7.0, both, were used as complexation media. The resulting suspensions were incubated for 30 min at 37 °C prior to use.

Polyplexes were prepared by adding the aqueous solution of pDNA to the polymers in the appropriate complexation medium at a PEI:pDNA stoichiometry (v:v) of 10:1. To evaluate the influence of the mode of addition, reagents were added dropwise (referred to as DROPPING) or mixed by pipetting (referred to as MIXING). More into detail, DROPPING procedure was as follows: pDNA solution was dropped all at once (one single drop) into the diluted polymer solution and the resulting solution was left standing; instead, MIXING consisted in the direct addition of pDNA to the diluted polymer solution followed by prompt, vigorous mixing by pipetting up and down for 10 times and ca 5 sec in total.

The opposite order of addition (PEI added to DNA) was also investigated. In such a case, PEI:pDNA stoichiometry (v:v) was 1:10.

### Measurement of size and ζ-potential of polyplexes

The *D*_*H*_ and the *ζ*_*P*_ of the polyplexes were measured by DLS and laser Doppler micro-electrophoresis using a Malvern Zetasizer Nano ZS instrument (Malvern, UK), fitted with a 5 mW HeNe laser, 633 nm, at a fixed scattering angle of 173°. For each complexation condition, 50 μL of polyplexes containing 1 μg of DNA were prepared as described above, incubated for 30 min at 37 °C and next diluted 1:9 with the complexation buffer (10 mM HEPES buffer or 150 mM NaCl at pH 7.0, both). Measurements were performed 5 min after dilution. To investigate the influence of culture medium on the dimensions of polyplexes, 50 μL of complexes were diluted 1:9 with phenol red-free culture medium and the variation in their diameter was monitored over 4 hrs at 37 °C. Three different culture media were tested: DMEM supplemented with 1 mM sodium pyruvate, 10 mM HEPES buffer, 100 U/mL penicillin, 0.1 mg/mL streptomycin, 2 mM glutamine (referred to as serum-free DMEM), serum-free DMEM supplemented with 10% FBS (referred to as complete DMEM) and OptiMEM^®^ I (referred to as OptiMEM).

Autocorrelation functions were analysed using the standard cumulant method^21^, described in ISO 13321:1996 and ISO22412:2008, producing a mean value for the *D*_*H*_ (Z-Average) and a width parameter of the monomodal curve known as polydispersity index (PDI). Size distribution analysis, using a continuous non-negative least squares (NNLS) fit of the autocorrelation function (CONTIN algorithm^35^,^36^), was used to analyze polyplexes diluted in complete cell culture medium.

### Cell culture and transfection

Transfection studies were performed on HeLa cells at passage between 10 and 18 and on HUASMCs at passage always between 5 and 10. HeLa cells and HUASMCs were cultured at 37 °C in a humidified atmosphere and under constant supply of 5% (v/v) CO_2_, respectively in complete DMEM and in complete DMEM supplemented with 5 μg/mL human insulin, 2 ng/mL FGFb, and 0.5 ng/mL EGF. For all the experiments, cells were passaged 24 hrs before final seeding (2 × 10^4 ^cells/cm^2^ in T25 flasks) and then plated in 96-well plates at a density of 2 × 10^4^ cells/cm^2^ and maintained in complete DMEM. After 24 hrs, 320 ng of pDNA/cm^2^ for HeLa cells and 160 ng of pDNA/cm^2^ for HUASMCs were complexed (v/v) with 25 kDa bPEI or 25 kDa lPEI in 10 mM HEPES or 150 mM NaCl to give N/P 30, according to the different complexation protocols described herein above. Polyplexes were next incubated with cells for 24 hrs in 150 μL/cm^2^ of complete DMEM or OptiMEM. For the experiments in serum-free DMEM, cells were incubated with polyplexes for 4 hrs, then the medium was replaced with complete DMEM and the cells cultured for further 20 hrs.

For transfection experiments under increased gravitational load, plates were centrifuged for 5 min at 500 × g in a multipurpose centrifuge with swing bucket rotor and microplate adaptor (Eppendorf 5810 R, Eppendorf, Milan, Italy), immediately after addition of polyplexes to cells. Experiments were performed at least in quadruplicate.

### Cellular uptake experiments

**Figure 5:**
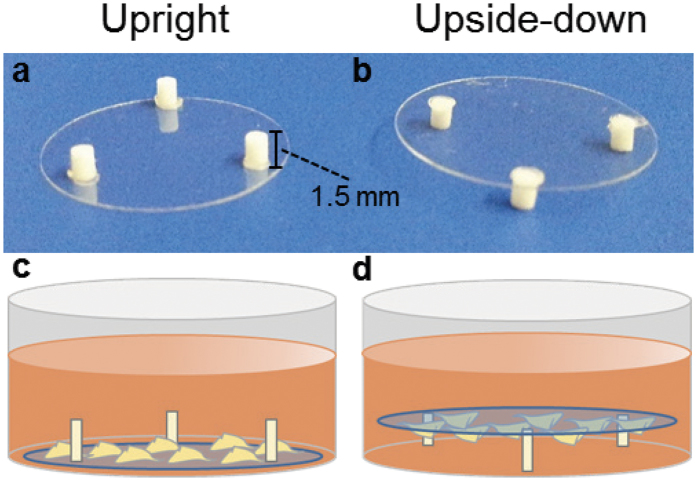
*Ad hoc*-prepared 18 mm-diameter glass coverslips featuring three polyethylene cylindrical spacers (1.5 mm high, 1 mm diameter) in (**a**) upright and (**b**) upside-down configurations. Schematic representation of the (**c**) upright and (**d**) upside-down experimental setups, showing cells facing the top and the bottom of the cell culture well, respectively.

Cell culture supports were prepared *ad hoc* for cellular uptake and subsequent upside-down transfection experiments. Briefly, three polyethylene cylindrical spacers (1.5 mm height, 1 mm diameter, Fig. 5) were glued on the surface of 18 mm diameter glass coverslips using Silastic^®^ Medical Adhesive Silicone Type A (Dow Corning, Auburn, MI, USA). After overnight incubation under vacuum to allow the glue to polymerize and dry, the coverslips were sterilized in pure ethanol (30 min) under UV irradiation (15 W germicidal lamp, 30 min per side).

Coverslips were placed upward in 12-well plates (with the spacers facing upward) and cells were seeded in the conditions described above. Twenty-four hrs after seeding, coverslips were moved to empty wells by means of sterile tweezers, and turned upside-down with spacers leant on the well surface and cells facing the bottom of the well (upside-down configuration, Fig. 5d). Coverslips seeded with cells and placed upright in empty wells were used as controls (Fig. 5c). Cellular uptake experiments consisted in the delivery of 1 mg/mL of FITC-labelled dextran solubilized in OptiMEM. Four hrs post-delivery at 37 °C, supernatants were discarded, cells were washed once in PBS, detached by trypsin, and pelleted. Proteins were solubilized in 50 mM HEPES pH 7.4, 1 mM PMSF, 2 μg/mL leupeptin, 2 μg/mL aprotinin, 1 μg/mL pepstatin, 1% (v/v) Triton X100 in PBS and protein content determined by BCA protein assay kit. Finally, the fluorescence was read by means of a spectrofluorimeter (λ_ex_ = 495 nm; λ_em_ = 521 nm).

### Upside-down transfection experiments

Coverslips were prepared and seeded as described herein above. Twenty-four hrs after seeding, coverslips were moved to empty wells, and partly turned upside-down, partly placed upright (controls) as described for cellular uptake experiments. Complete DMEM supplemented with pGL3/PEI or pEGFP/PEI polyplexes was next added to cells. For fluorescence microscopy analysis, the spacers were removed and cells fixed in 3.7% (w/v) paraformaldehyde in deionized water (dH_2_O), permeabilized with 0.5% (v/v) Triton X100 in PBS and nuclei stained with 0.3 μg/mL of DAPI. Coverslips were subsequently mounted on glass slides and digital images were obtained by means of an Olympus BX51 fluorescence microscope (Olympus, Tokyo, Japan).

### Evaluation of cytotoxicity

Twenty-four hrs after transfection, cytotoxicity of polyplexes was assessed by AlamarBlue cell viability assay used in accordance to the manufacturer’s guidelines. Briefly, medium was replaced with complete DMEM containing 1× resazurin dye and cells were incubated in standard culture conditions. After two hrs, the fluorescence of the cell culture medium was measured by means of a GENios Plus reader (λ_ex_ = 560 nm; λ_em_ = 590 nm). Viability of untreated controls was assigned to as 100% and cytotoxicity was determined according to the following equation:


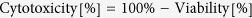


### Evaluation of transfection efficiency

Transgene activity was measured by Luciferase Assay System as previously described. Briefly, cells were washed with PBS, lysed with Cell Culture Lysis Reagent (Promega Italia, Milan, Italy) and freeze-thawed once to facilitate cell disruption. Twenty μL of cell lysate were next mixed with 50 μL of Luciferase Assay Reagent and luminescence was measured by means of a GENios Plus reader. The luminescence signal of each sample was normalized to its protein content, determined using BCA assay and data were finally expressed as relative light units per mg of proteins (RLU/mg of proteins).

### Statistical analysis

Statistical analysis was carried out by GraphPad version 5.04 (GraphPad software, La Jolla, CA, USA). Comparisons among groups were performed by one-way analysis of variance (ANOVA) and multiple t-test with Sidak-Bonferroni adjustment for multiple testing. Significance was retained when *p* < 0.05.

## Additional Information

**How to cite this article**: Pezzoli, D. *et al*. Size matters for *in vitro* gene delivery: investigating the relationships among complexation protocol, transfection medium, size and sedimentation. *Sci. Rep.* **7**, 44134; doi: 10.1038/srep44134 (2017).

**Publisher's note:** Springer Nature remains neutral with regard to jurisdictional claims in published maps and institutional affiliations.

## Supplementary Material

Supplementary Information

## Figures and Tables

**Table 1:** Hydrodynamic diameter (*D*_*H*_), polydispersity index (PDI) and ζ-potential (*ζ*_*P*_) of the polyplexes were measured by dynamic light scattering (DLS) and laser Doppler micro-electrophoresis.

			*D*_*H*_ (nm)	St. Dev. *D*_*H*_ (nm)	PDI	St. Dev. PDI	*ζ*_*P*_ (mV)	St. Dev. *ζ*_*P*_ (mV)
**25 kDa bPEI**	10 mM Hepes	MIXING	143.8	6.4	0.16	0.03	30.2	0.9
		DROPPING	386.5	38.7	0.41	0.08	32.2	1.1
	150 mM NaCl	MIXING	159.8	10.9	0.18	0.05	22.1	1.1
		DROPPING	352.0	34.0	0.50	0.17	28.7	3.1
**25** **kDa lPEI**	10 mM Hepes	MIXING	161.4	12.9	0.19	0.07	24.5	1.9
		DROPPING	466.0	63.8	0.44	0.07	31.0	0.8
	150 mM NaCl	MIXING	2296.2	255.4	1.00	0.00	27.1	1.0
		DROPPING	3832.9	1209.9	0.96	0.11	22.5	1.3

25 kDa bPEI- and lPEI-based polyplexes were prepared at N/P 30 by adding pDNA to PEI solution, in 10 mM HEPES at pH 7 or 150 mM NaCl at pH 7. Reagents were added dropwise (DROPPING mode) or mixed by pipetting (MIXING mode). Measurements were performed 5 min after dilution in buffer (n = 4).
