# Supplementary material for: Size matters for in vitro gene delivery: investigating the relationships among complexation protocol, transfection medium, size and sedimentation
Source: Sci Rep. 2017 Mar 8;7:44134. doi: 10.1038/srep44134 (PMC5341125; doi:10.1038/srep44134)
Supplement: Supplementary Information [file srep44134-s1.doc]

**Supplementary information**

**Size matters for *in vitro* gene delivery: investigating the relationships among complexation protocol, transfection medium, size and sedimentation**

**Daniele Pezzoli^1^*^,3^,* Elisa Giupponi*^2^*, Diego Mantovani^3^, Gabriele Candiani^1^,*^2*^***

^1^ Research Unit Milano Politecnico, National Interuniversity Consortium of Materials Science and Technology – INSTM, Via Mancinelli 7, Milan 20131, Italy

^2^ Department of Chemistry, Materials and Chemical Engineering “Giulio Natta”, Politecnico di Milano, Via Mancinelli 7, Milan 20131, Italy

^3^ Laboratory for Biomaterials and Bioengineering, CRC-I, Department of Mining, Metallurgical and Materials Engineering & CHU de Quebec Research Centre, Laval University, 10 rue de l'Espinay, Quebec City (QC) G1L 3L5, Canada

* Corresponding Author:

Gabriele Candiani, Ph.D.

Department of Chemistry, Materials and Chemical Engineering Politecnico di Milano

Via Mancinelli 7, Milan 20131, Italy

[gabriele.candiani@polimi.it](mailto:gabriele.candiani@polimi.it)

Tel.: +390223993181

Fax: +390223993180

Supplementary information

Contents

*Supplementary Table S1 2*

*Supplementary Figure S1 3*

*Supplementary Figure S2 4*

*Supplementary Figure S3 5*

*Supplementary Figure S4 6*

*Supplementary Figure S5 7*

*Supplementary Figure S6 8*

*Supplementary Figure S7 9*

*Supplementary Figure S8 10*

*Supplementary Figure S9 11*

*Supplementary Figure S10 12*

*Supplementary Figure S11 13*

**Supplementary Table S1**

**Supplementary Table S1.** Hydrodynamic diameter (*D_H_*), polydispersity index (PDI) and ζ-potential (*ζ_P_*) of the polyplexes measured by dynamic light scattering (DLS) and laser Doppler micro-electrophoresis. 25 kDa bPEI- and lPEI-based polyplexes were prepared in 10 mM HEPES and 150 mM NaCl, invariably at N/P 30, by adding PEI to pDNA solution. Reagents were added dropwise (DROPPING mode) or mixed by pipetting (MIXING mode). Measurements were performed 5 min after dilution in buffer (n = 4).

**Supplementary Figure S1**


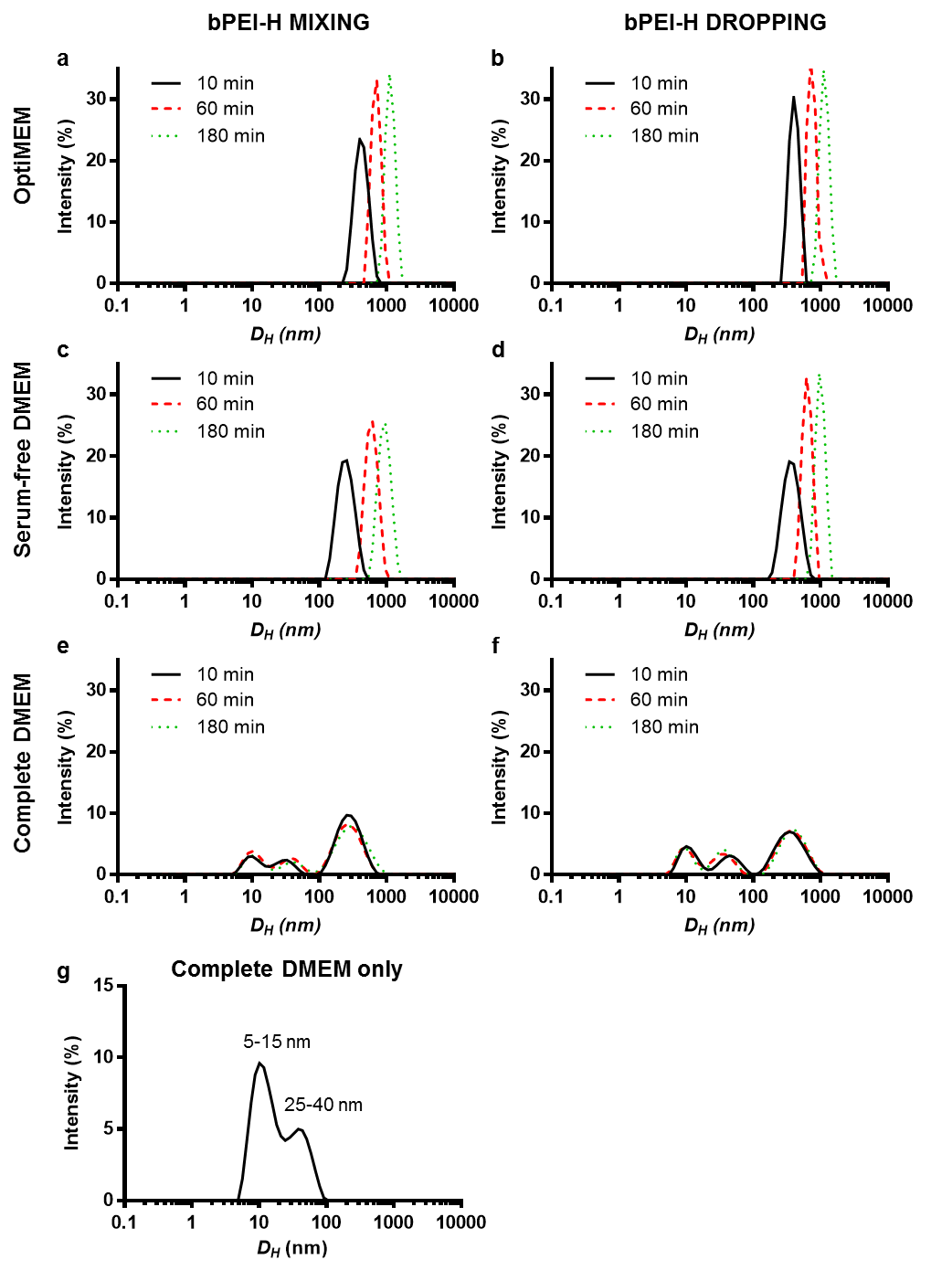


**Supplementary Figure S1.** (a-f) Representative DLS spectra (multimodal size distribution analysis) of bPEI polyplexes prepared by adding pDNA to bPEI solution in 10 mM HEPES according to the (a, c, e) MIXING or (b, d, f) DROPPING protocol and next diluted in (a, b) OptiMEM, (c, d) serum-free DMEM and (e, f) complete DMEM. Size distribution plots were acquired 10 (black curve), 60 (red dashed curve) and 180 (green dotted curve) min after dilution in culture media. (g) Representative DLS spectra of complete DMEM.

**Supplementary Figure S2**


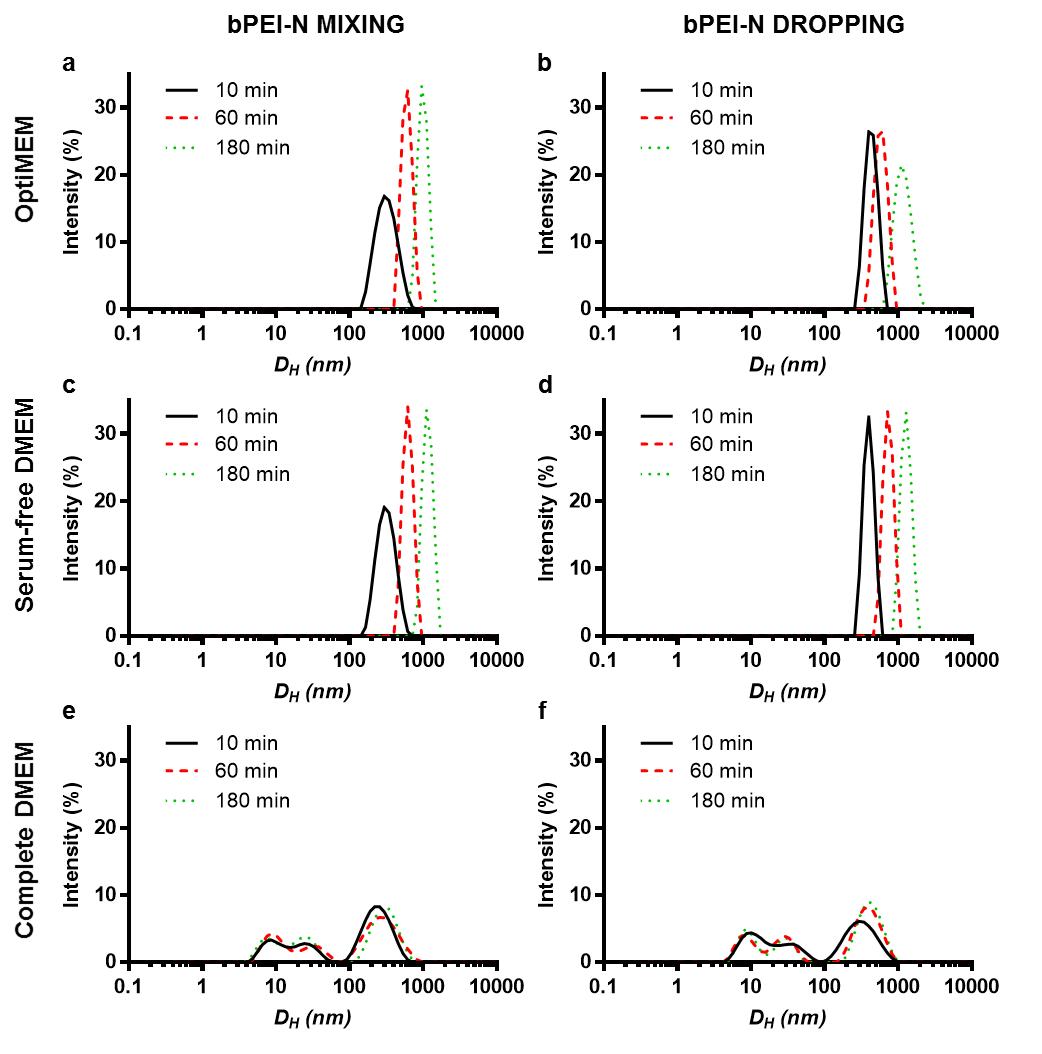


**Supplementary Figure S2.** Representative DLS spectra (multimodal size distribution analysis) of bPEI polyplexes prepared by adding pDNA to bPEI solution in 150 mM NaCl according to the (a, c, e) MIXING or (b, d, f) DROPPING protocol and next diluted in (a, b) OptiMEM, (c, d) serum-free DMEM and (e, f) complete DMEM. Size distribution plots were acquired 10 (black curve), 60 (red dashed curve) and 180 (green dotted curve) min after dilution in culture media.

**Supplementary Figure S3**


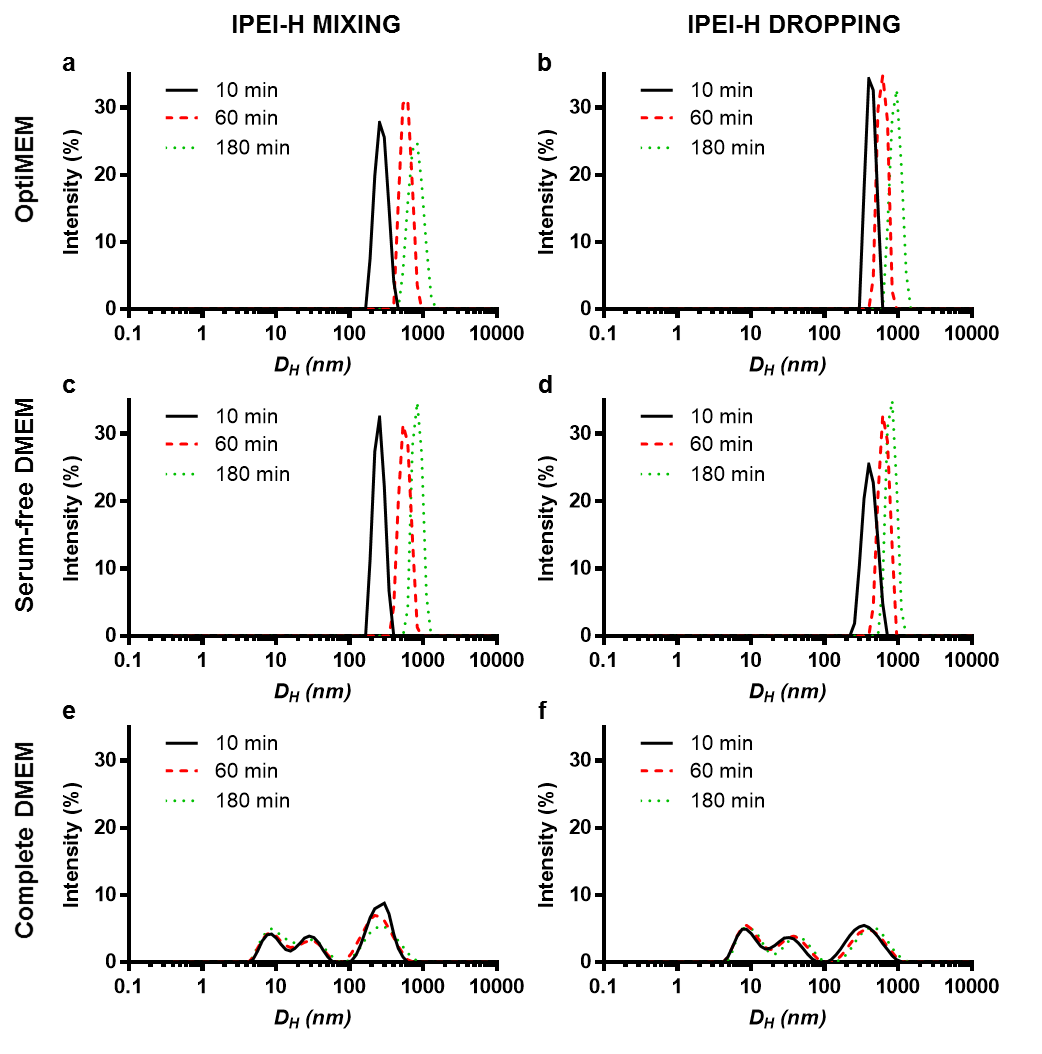


**Supplementary Figure S3.** Representative DLS spectra (multimodal size distribution analysis) of lPEI polyplexes prepared by adding pDNA to lPEI solution in 10 mM HEPES according to the (a, c, e) MIXING or (b, d, f) DROPPING protocol and next diluted in (a, b) OptiMEM, (c, d) serum-free DMEM and (e, f) complete DMEM. Size distribution plots were acquired 10 (black curve), 60 (red dashed curve) and 180 (green dotted curve) min after dilution in culture media.

**Supplementary Figure S4**

**
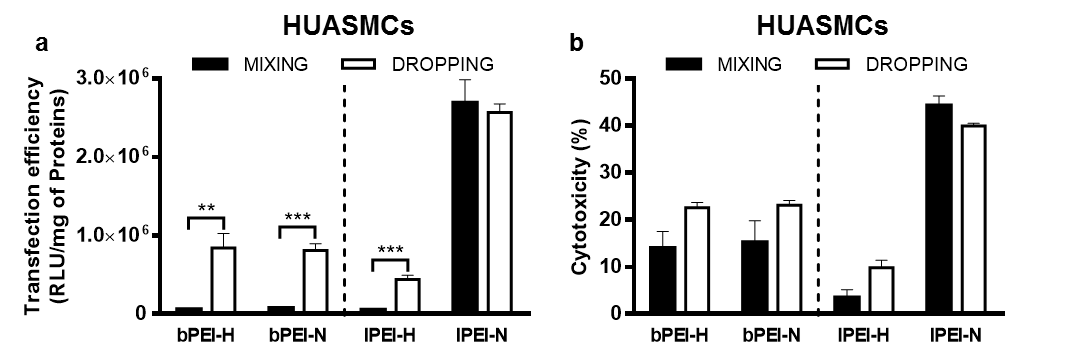
**

**Supplementary Figure S4.** (a) Transfection efficiency and (b) cytotoxicity of polyplexes prepared according to different complexation protocols and tested in complete DMEM on HUASMCs. Polyplexes were prepared in 10 mM HEPES (bPEI-H and lPEI-H) and 150 mM NaCl (bPEI-N and lPEI-N) at N/P 30 according to the MIXING and DROPPING modes, invariably adding pDNA to PEI solution. After dilution in complete DMEM, polyplexes were added to cells and transfection efficiency and cytotoxicity were evaluated at 24 hrs. Results are expressed as mean ± SD, n = 4. **: *p* < 0.01, ***: *p* < 0.001, MIXING vs. DROPPING.

**Supplementary Figure S5**


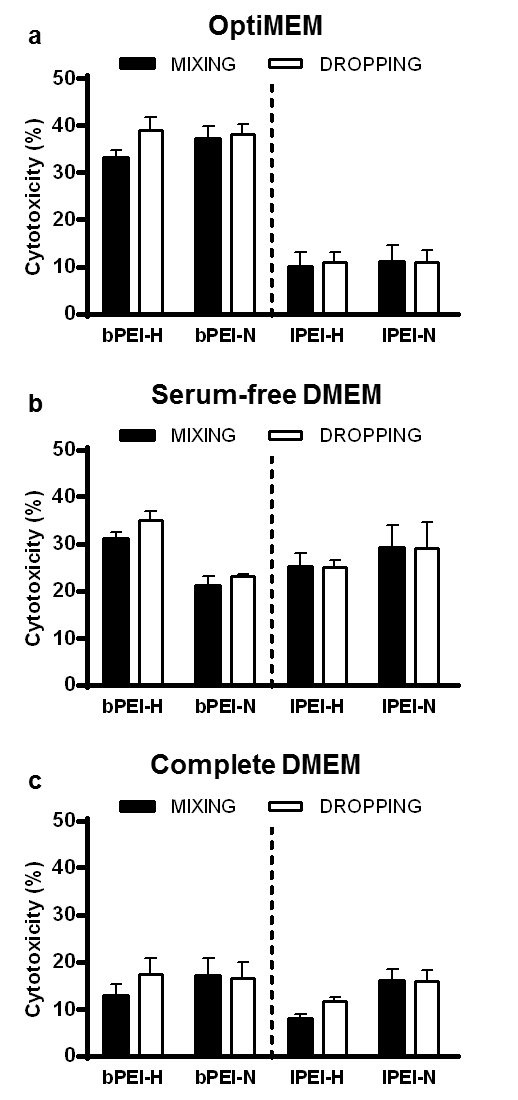


**Supplementary Figure S5.** Cytotoxicity of polyplexes prepared according to different complexation protocols and tested in various transfection media. Polyplexes were prepared in 10 mM HEPES (bPEI-H and lPEI-H) and 150 mM NaCl (bPEI-N and lPEI-N) at N/P 30 according to the MIXING and DROPPING modes, invariably adding pDNA to PEI solution. After dilution in (a) OptiMEM, (b) serum-free DMEM and (c) complete DMEM, polyplexes were added to HeLa cells and cytotoxicity was evaluated 24 hrs post-delivery. Results are expressed as mean ± SD, n ≥ 8.

**Supplementary Figure S6**


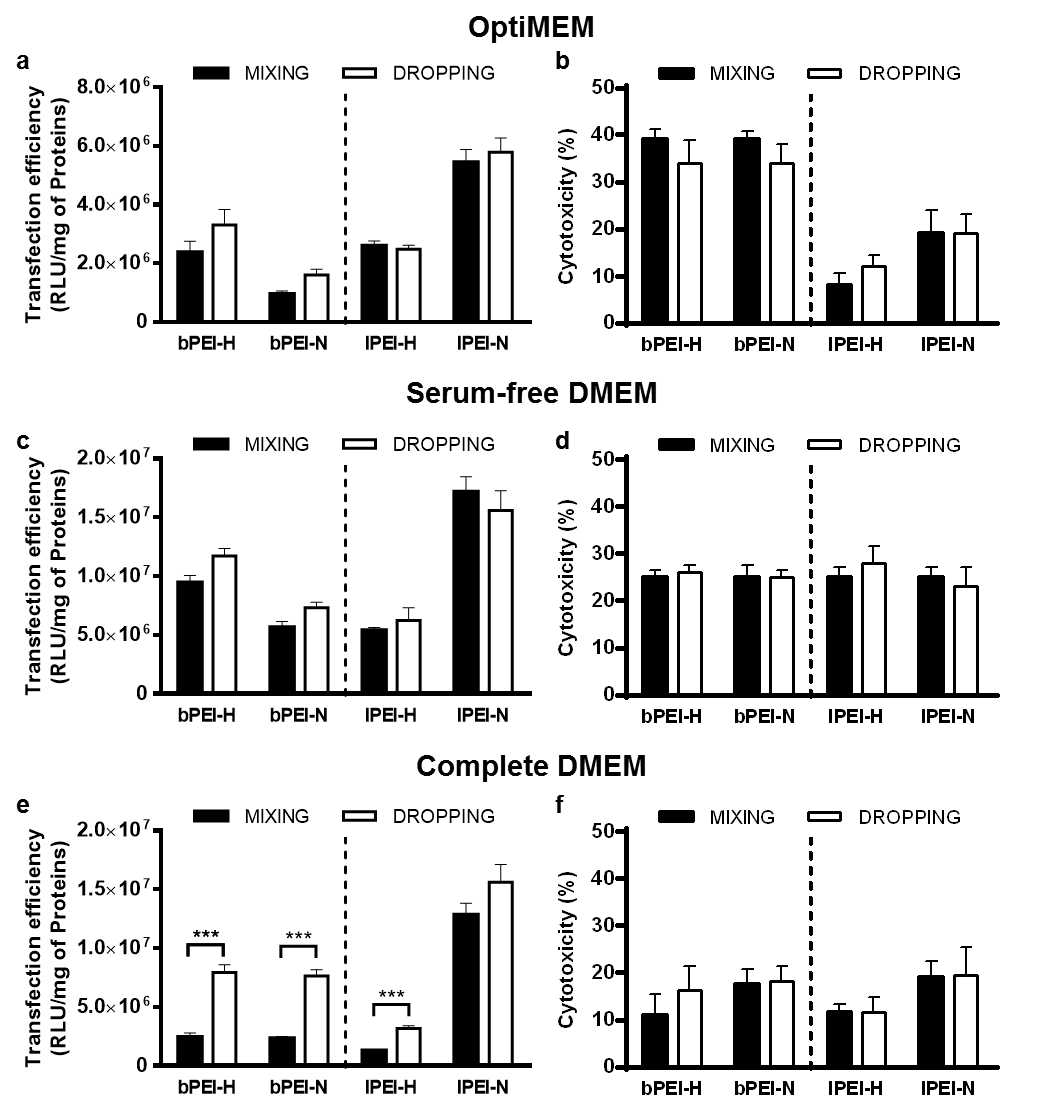


**Supplementary Figure S6.** (a, c, e) Transfection efficiency and (b, d, f) cytotoxicity of bPEI and lPEI polyplexes prepared by adding PEI to pDNA solution according to different complexation protocols and tested in different transfection media. Polyplexes were prepared in 10 mM HEPES (bPEI-H and lPEI-H) and 150 mM NaCl (bPEI-N and lPEI-N) at N/P 30 according to the MIXING and DROPPING modes, invariably adding PEI to pDNA solution. Polyplexes were added to HeLa cells in (a, b) OptiMEM, (c, d) serum-free DMEM or (e, f) complete DMEM and transfection efficiency and cytotoxicity were evaluated 24 hrs post-delivery. Results are expressed as mean ± SD, n ≥ 8. ***: *p* < 0.001, MIXING *vs.* DROPPING.

**Supplementary Figure S7**

**Supplementary Figure S7.** Viability of untreated HeLa control cells. Cells were seeded at a density of 2 × 10^4^ cells/cm^2^ and maintained in complete DMEM. After 24 hrs medium was changed with complete DMEM, serum-free DMEM or OptiMEM. Serum-free DMEM was replaced with complete DMEM after 4 hrs. Viability was evaluated 24 hrs post medium change (48 hrs post seeding) by AlamarBlue cell viability assay. The fluorescence of the resazurin dye-containing medium after 2 hrs of incubation is reported. Results are expressed as mean ± SD, n ≥ 8. *: *p* < 0.05.

**Supplementary Figure S8**


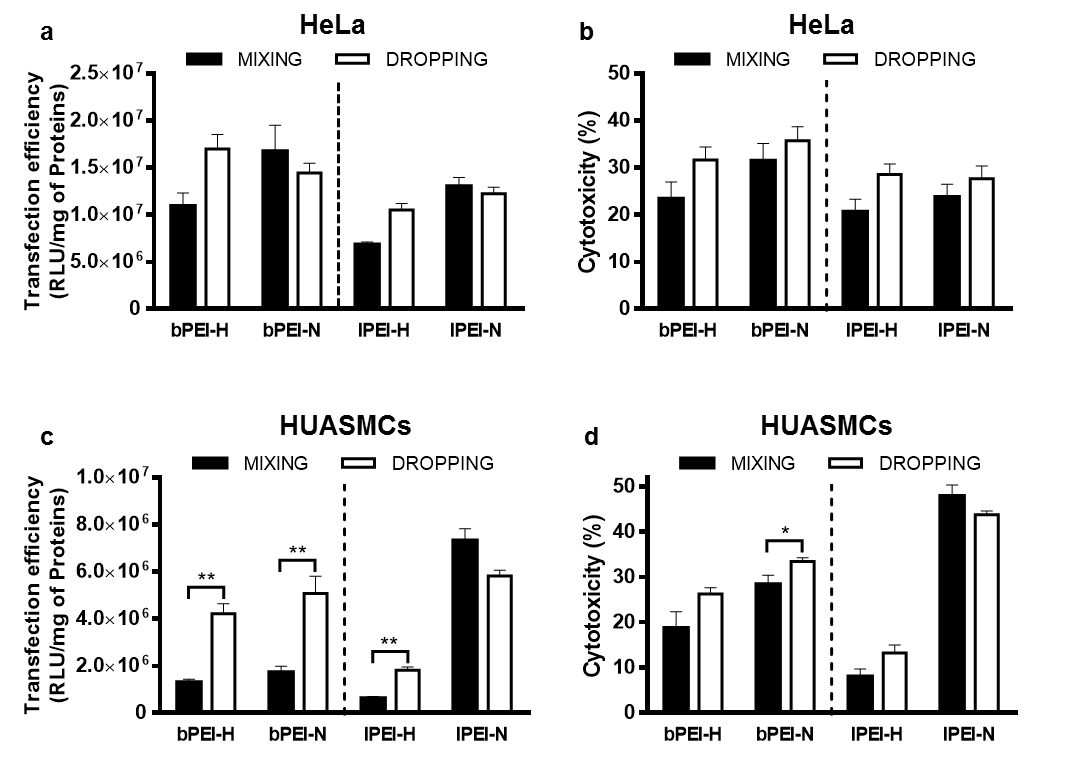


**Supplementary Figure S8.** (a, c) Transfection efficiency and (b, d) cytotoxicity of bPEI and lPEI polyplexes upon centrifugation. Polyplexes were prepared in 10 mM HEPES (bPEI-H and lPEI-H) and 150 mM NaCl (bPEI-N and lPEI-N) at N/P 30 according to the MIXING and DROPPING modes, invariably adding pDNA to PEI solution. Centrifugations consisted in the addition of polyplexes to (a, b) HeLa cells and (c, d) HUASMCs in complete DMEM, followed by prompt centrifugation for 5 min at 500×g. Transfection efficiency and cytotoxicity were evaluated 24 hrs post-delivery. Results are expressed as mean ± SD, n ≥ 4. *: *p* < 0.05, **: *p* < 0.01, MIXING vs. DROPPING.

**Supplementary Figure S9**

**Supplementary Figure S9.** Cellular uptake of 70 kDa FITC-labelled dextran. HeLa cells were held upright and upside-down during the delivery step (4 hrs). Results are expressed as mean ± SD, n = 3.

**Supplementary Figure S10**


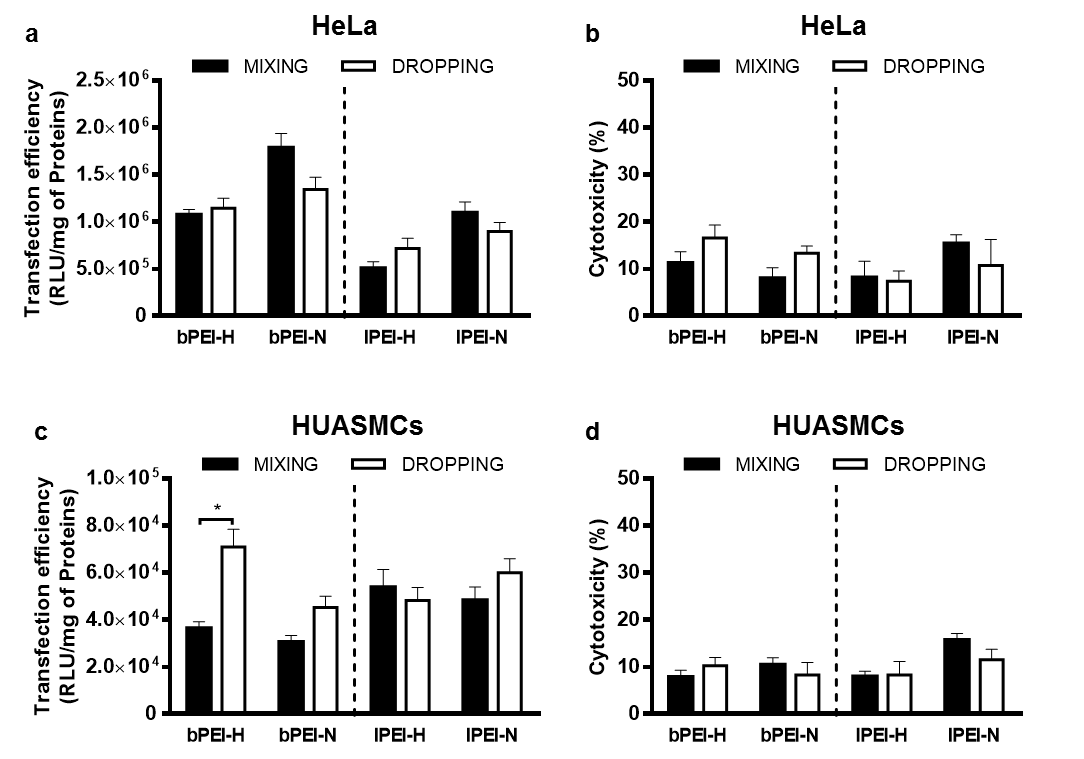


**Supplementary Figure S10.** (a, c) Transfection efficiency and (b, d) cytotoxicity for experiments in the upside-down configuration. Polyplexes were prepared in 10 mM HEPES (bPEI-H and lPEI-H) and 150 mM NaCl (bPEI-N and lPEI-N) at N/P 30 according to the MIXING and DROPPING modes, invariably adding pDNA to PEI solution. Polyplexes were mixed with complete DMEM and added to (a, b) HeLa cells and (c, d) HUASMCs held upside-down. Transfection efficiency and cytotoxicity were evaluated 24 hrs post-delivery. Results are expressed as mean ± SD, n ≥ 8. * *p* < 0.05, MIXING *vs.* DROPPING.

**Supplementary Figure S11**

**
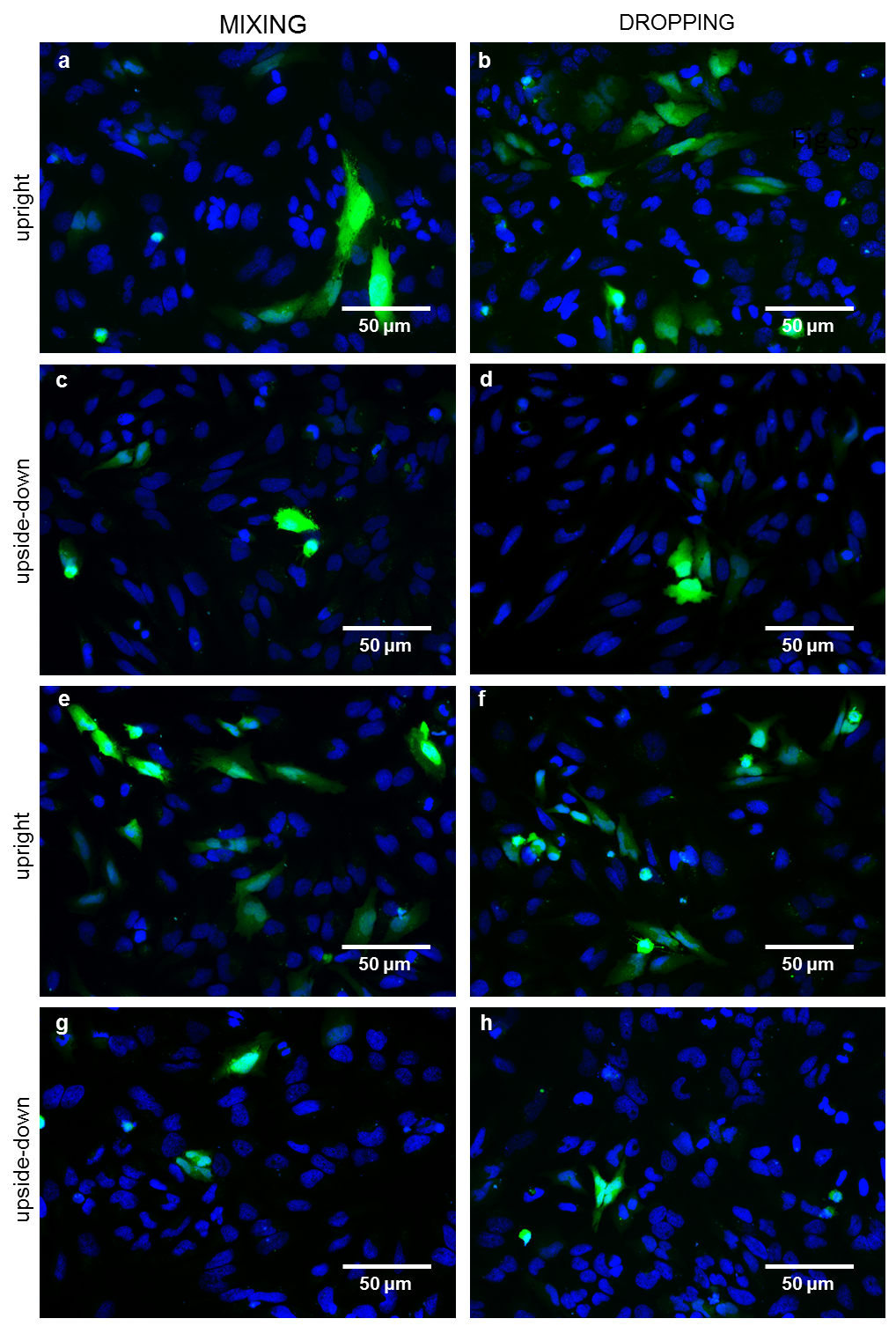
**

**Supplementary Figure S11.** Transfection experiments in upside-down configuration using pEGFP-N1 plasmid. (a-d) bPEI and (e-h) lPEI polyplexes were prepared in 10 mM HEPES and 150 mM NaCl, respectively, invariably at N/P 30 adding pDNA to PEI solution according to the (a, c, e, g) MIXING and (b, d, f, h) DROPPING modes. Polyplexes were added to HeLa cells cultured upright (a, b, e, f) and (c, d, g, h) upside-down in complete DMEM. pEGFP expression (green fluorescence) was evaluated 24 hrs post-delivery. Cell nuclei were stained with DAPI (blue).
